# Oocytes Selected Using BCB Staining Enhance Nuclear Reprogramming and the In Vivo Development of SCNT Embryos in Cattle

**DOI:** 10.1371/journal.pone.0036181

**Published:** 2012-04-27

**Authors:** Jianmin Su, Yongsheng Wang, Ruizhe Li, Hui Peng, Song Hua, Qian Li, Fusheng Quan, Zekun Guo, Yong Zhang

**Affiliations:** 1 College of Veterinary Medicine, Northwest A & F University, Key Laboratory of Animal Reproductive Physiology & Embryo Technology, Ministry of Agriculture, Yangling, Shaanxi, People's Republic of China; 2 Department of Biochemistry and Molecular Biology, College of Life Sciences, Northwest A & F University, Yangling, Shaanxi, People's Republic of China; University of Connecticut, United States of America

## Abstract

The selection of good quality oocytes is crucial for *in vitro* fertilization and somatic cloning. Brilliant cresyl blue (BCB) staining has been used for selection of oocytes from several mammalian species. However, the effects of differential oocyte selection by BCB staining on nuclear reprogramming and *in vivo* development of SCNT embryos are not well understood. Immature compact cumulus–oocyte complexes (COCs) were divided into control (not exposed to BCB), BCB+ (blue cytoplasm) and BCB− (colorless cytoplasm) groups. We found that BCB+ oocytes yielded a significantly higher somatic cell nuclear transfer (SCNT) blastocyst rate and full term development rate of bovine SCNT embryos than the BCB− and control oocytes. BCB+ embryos (embryos developed from BCB+ oocytes) showed increased acetylation levels of histone H3 at K9 and K18 (AcH3K9, AcH3K18), and methylation levels of histone H3 at K4 (H3K4me2) than BCB− embryos (embryos developed from BCB− oocytes) at the two-cell stage. Furthermore, BCB+ embryos generated more total cells, trophectoderm (TE) cells, and inner cell mass (ICM) cells, and fewer apoptotic cells than BCB− embryos. The expression of *SOX2*, *CDX2*, and anti-apoptotic microRNA-21 were up-regulated in the BCB+ blastocysts compared with BCB− blastocysts, whereas the expression of pro-apoptotic gene *Bax* was down-regulated in BCB+ blastocysts. These results strongly suggest that BCB+ oocytes have a higher nuclear reprogramming capacity, and that BCB staining can be used to select developmentally competent oocytes for nuclear transfer.

## Introduction

The mammalian oocyte not only has the unique ability to support fertilization in normal development, but also has the capacity to reprogram nuclei of somatic cells toward pluripotency. Oocyte quality has been shown to contribute to poor somatic cloning efficiency with many factors affecting oocyte quality and developmental potential. The follicular oocytes used in somatic cell nuclear transfer (SCNT) are commonly recovered from ovaries of slaughtered cattle of unknown age, breed, health status and reproductive performance, and are therefore heterogeneous in quality and developmental competence [1]. Generally, oocytes are selected using morphological assessment by observing the numbers and compactness of cumulus cell layers surrounding the oocyte, granulation and homogeneity of the cytoplasm. However, the performance of oocytes selected using these vague criteria is often conflicting and inaccurate, making it difficult to distinguish oocytes of developmental competence. Therefore, finding a non-invasive and non-perturbing method for selection of oocytes prior to culture has become of prime importance.

It is generally believed that glucose-6-phosphate dehydrogenase (G6PDH) protein is active in the growing oocyte, but its activity is decreased in oocytes that have finished their growth phase, and are then likely to have achieved developmental competence. The enzyme G6PDH can degrade brilliant cresyl blue (BCB). Thus, oocytes yielding decreased G6PDH (finished growth phase) show a blue cytoplasm (BCB+) after BCB staining, while growing oocytes (unfinished growth phase) have abundant G6PDH and a colorless cytoplasm (BCB−). BCB staining has been used for the selection of oocytes from several mammalian species. It was reported that BCB+ oocytes yielded a significantly higher blastocyst developmental rate than the BCB− and control oocytes in pig [2], goat [3], [4], sheep [5], mouse [6], dog [7], and bovine [1], [8]–[10]. However, whether BCB+ oocytes enhance the *in vivo* and full-term development of SCNT embryos and why BCB+ oocytes (with finished growth) are better than BCB- (growing oocytes) or non-treated oocytes (control oocytes) is unknown.

Here, we explored the *in vivo* and full-term developmental competence of bovine SCNT embryos derived from differential oocytes (BCB+, BCB−, and non-treated oocytes) to investigate whether BCB+ oocytes could yield higher cloning efficiency. To investigate why BCB+ oocytes are better than BCB− or non-treated oocytes and how cloning efficiency is improved, we analyzed the global acetylation levels of histone H3 at lysine 9 (AcH3K9) and 18 (AcH3K18) and global levels of histone H3 dimethylated at lysine 4 (H3K4me2) and 9 (H3K9me2) by immunostaining of SCNT embryos developed from BCB+ oocytes (BCB+ group), BCB− oocytes (BCB− group), and non-treated oocytes (control group). The total, trophectoderm (TE) and inner cell mass (ICM) cell numbers in blastocysts, the ratio of ICM: TE, and the rate of apoptosis in blastocysts were also measured by immunostaining and TUNEL assay to assess the quality of bovine SCNT embryos from the three groups. Furthermore, we compared the mRNA and microRNA levels of blastocysts from the three groups using quantitative real-time PCR and Taqman real-time PCR.

## Results

### Experiment 1: BCB+ oocytes support higher developmental competence of bovine cloned embryos *in vitro*

**Figure 1:**
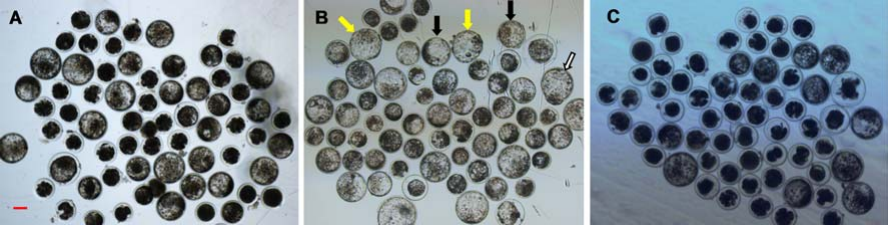
Representative photographs of bovine blastocysts. Day 7 SCNT blastocysts developed from control oocytes (A: control group), BCB+ oocytes (B: BCB+ group), and BCB− oocytes (C: BCB− group). Original magnification was ×40. Bar = 100 µm. Expanded, hatching, and hatched Blastocysts were directed by black, white, and yellow arrows, respectively.

Maturation rates of oocytes and developmental rates (to cleavage and day 7 blastocyst stage) of cloned embryos were analyzed from 50 replicates (Table 1, and Fig. 1). Maturation rate of oocytes and cleavage rate of SCNT embryos were higher in the BCB+ group compared with the BCB− group (P<0.05), but did not differ between the BCB+ and control groups (P>0.05). Blastocyst rate of SCNT embryos in the BCB+ group was significantly higher than those in the BCB− and control groups (P<0.05).

**Table 1:** Effect of oocyte selection by BCB staining on the development of bovine cloned embryos *in vitro*.

Group	No. oocytes	No. (%) MII oocytes	No. SCNT embryos cultured	No. (%) cleaved embryos	No. (%) blastocysts
Control	931	671 (72.37±2.21)^a^	456	316 (69.23±1.91)^a^	132 (28.64±0.72)^a^
BCB+	1480	1110 (75.13±2.03)^a^	775	550 (71.08±1.14)^a^	302 (39.03±0.44)^b^
BCB−	946	480 (50.63±3.21)^b^	288	138 (47.84±1.68)^b^	35 (12.11±2.32)^c^

50 replicates were performed. Maturation rate, cleavage rate, and blastocysts were showed as mean ± SEM%. Maturation rate: No. MII oocytes/No. oocytes. Cleavage rate: No. cleaved embryos/No. SCNT embryos cultured. Blastocyst rate: No. blastocysts/No. SCNT embryos cultured. Cleavage and blastocyst rates were monitored at 48 and 168 h of culture, respectively (0 h being the time embryos were transferred to G1.5).

a, b, c Values with different superscripts within columns are significantly different from each other (P<0.05).

### Experiment 2. BCB+ oocytes support higher developmental competence in bovine cloned embryos *in vivo*

A total of 68, 82, and 33 blastocysts from control, BCB+, and BCB− groups, respectively, were individually transferred to recipient Angus cows (Table 2). There was no significant difference between the BCB+ and control groups in the pregnancy rate at day 40. However, pregnancy rates from day 90 were significantly higher in the BCB+ group compared with the control and BCB− groups (P<0.05). Two calves were born from 68 recipients in the control group, whereas 7 calves were born from 82 recipients in the BCB+ group (P<0.05). No calves were produced from the BCB− group.

**Table 2:** BCB+ oocytes support higher developmental competence of bovine cloned embryos *in vivo*.

Groups	No. recipients	No. (%) pregnancies			No. (%) calves born
		Day 40	Day 90	Day 180	
Control	68	25 (36.89±4.46)^a^	5 (7.31±1.38)^a^	4 (5.86±1.42)^a^	2 (2.96±1.48)^a^
BCB+	82	31 (37.01±7.50)^a^	21 (24.96±6.12)^b^	13 (15.95±1.50)^b^	7 (8.69±1.67)^b^
BCB−	33	5 (15.15±3.03)^b^	1 (3.03±3.03)^a^	0 (0.00±0.00)^c^	0 (0.00±0.00)^a^

Three replicates were performed. The numbers of replicates were 22, 23, and 23 in control group, 25, 27, and 30 in BCB+ group, 11, 11, and 11 in BCB− group, respectively.

Day-7 blastocysts were non-surgically transferred (one embryo per recipient) to synchronized recipient cows.

a, b, c Values with different superscripts within columns are significantly different from each other (P<0.05).

### Experiment 3. Histone modifications were different in SCNT embryos from the three groups

**Figure 2:**
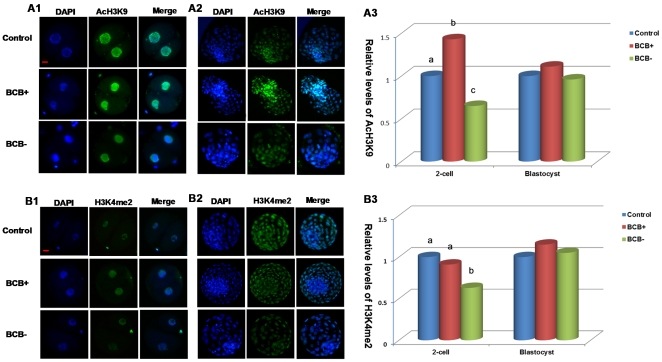
Global acetylation levels of H3K9 and global methylation levels of H3K4 in SCNT embryos. Staining of AcH3K9 (A) and H3K4me2 (B) in control, BCB+, and BCB− embryos at the two-cell (A1 for AcH3K9 and B1 for H3K4me2) and blastocyst stage (A2 for AcH3K9 and B2 for H3K4me2). Each sample was counterstained with DAPI to visualize DNA (blue). Original magnification was ×200. Bar = 20 µm. Quantification of AcH3K9/DNA (A3) and H3K4me2/DNA (B3) signal intensities in control (blue bars), BCB+ (red bars), and BCB− (green bars) embryos at the two-cell and blastocyst stage. Labeling intensity was expressed relative to that of the control embryos (set as 100%). Quantification of the AcH3K9/DNA and H3K4me2/DNA ratio is represented as the mean ± SEM. Values with different superscripts differ significantly (P<0.05). The experiments were replicated three times. In each replication, n = 10–15 per group.

To determine why the BCB+ oocyte yielded higher developmental potential of SCNT embryos, the global acetylation levels of H3K9 and H3K18, and the global methylation levels of H3K4 and H3K9 were measured in two-cell and blastocyst stage embryos (Fig. 2). No signals were detected in the embryos stained without primary or secondary antibodies, indicating the specificity of staining of the primary antibody. The levels of AcH3K9 (Fig. 2A, panel 1, and Fig. 2A, panel 3), AcH3K18, and H3K4me2 (Fig. 2B, panel1, and 2B, panel 3) in BCB+ embryos at the two-cell stage were higher than in BCB− embryos. There was a similar level of AcH3K9 (Fig. 2A, panel 2, and 2A, panel 3) and H3K4me2 (Fig. 2B, panel 2, and 2B, panel 3) among the three groups at the blastocyst stage, but the acetylation level of H3K18 in BCB+ embryos at blastocyst stage was higher than in BCB− and control embryos. No differences in H3K9me2 levels were observed among the groups.

### Experiment 4. Differential oocytes selected by BCB staining and the quality of cloned embryos: Number of total, ICM, and TE cells, and the ICM: TE ratio of SCNT blastocysts

**Figure 3:**
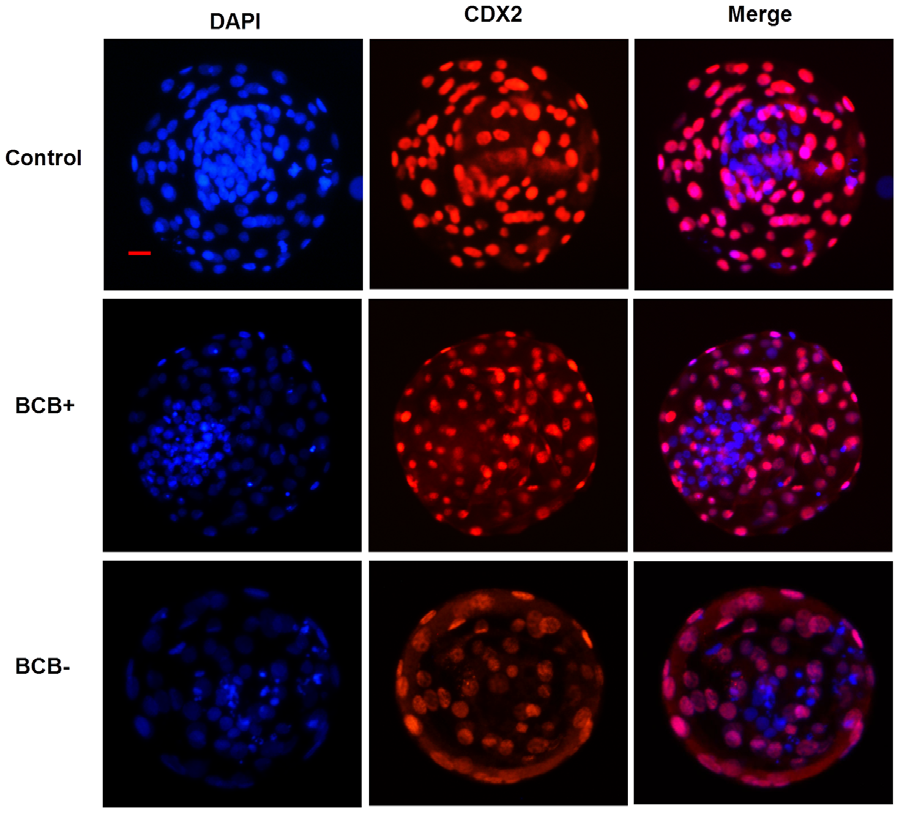
Immunostaining of CDX2. Day 7 SCNT blastocysts developed from control oocytes (control group), BCB+ oocytes (BCB+ group), and BCB− oocytes (BCB− group) were stained with DAPI and CDX2, a marker for trophectoderm. Original magnification was ×200. Bar = 20 µm. n = 35, 36, and 22 in the control, BCB+, and BCB− groups, respectively.

To determine if the improved *in vivo* development of SCNT embryos is reflected in blastocyst quality, the total cells, TE cells, and ICM cells were quantitated, and the ICM: TE ratio was calculated for SCNT blastocysts. As shown in Table 3 and Figure 3, total cell number, TE cell number, ICM cell number, and the ICM: TE ratio of blastocysts were significantly higher in the BCB+ group compared with the BCB− group (P<0.05). The total number of blastomeres and the ICM: TE ratio was also significantly higher in the BCB+ blastocysts than in the control blastocysts (P<0.05).

**Table 3:** Characterization of day 7 bovine blastocysts of three groups.

Groups	No. blastocyst analyzed	Total no. of cells	No. of TE cells	No. of ICM cells	ICM: TE (%)
Control	35	108.47±9.14^a^	82.13±6.75^a^	26.33±2.64^ab^	31.98±1.48^a^
BCB+	36	130.68±6.34^b^	88.68±4.26^a^	42.00±2.26^a^	47.31±1.21^b^
BCB−	22	70.95±5.83^c^	54.68±4.12^b^	16.26±1.89^b^	29.06±1.39^a^

The cell numbers in blastocysts were estimated by counting the total number of nuclei using DAPI, and the number of trophectoderm (TE) nuclei was estimated using immunostaining for CDX2. The cell number of the ICM was assessed as the total number of nuclei minus the number of TE nuclei. The data were shown as Mean ± SEM.

a, b, c Values with different superscripts within columns are significantly different from each other (P<0.05).

### Experiment 5. Differential oocytes selected by BCB staining and the quality of cloned embryos: Apoptotic index of SCNT blastocysts

**Figure 4:**
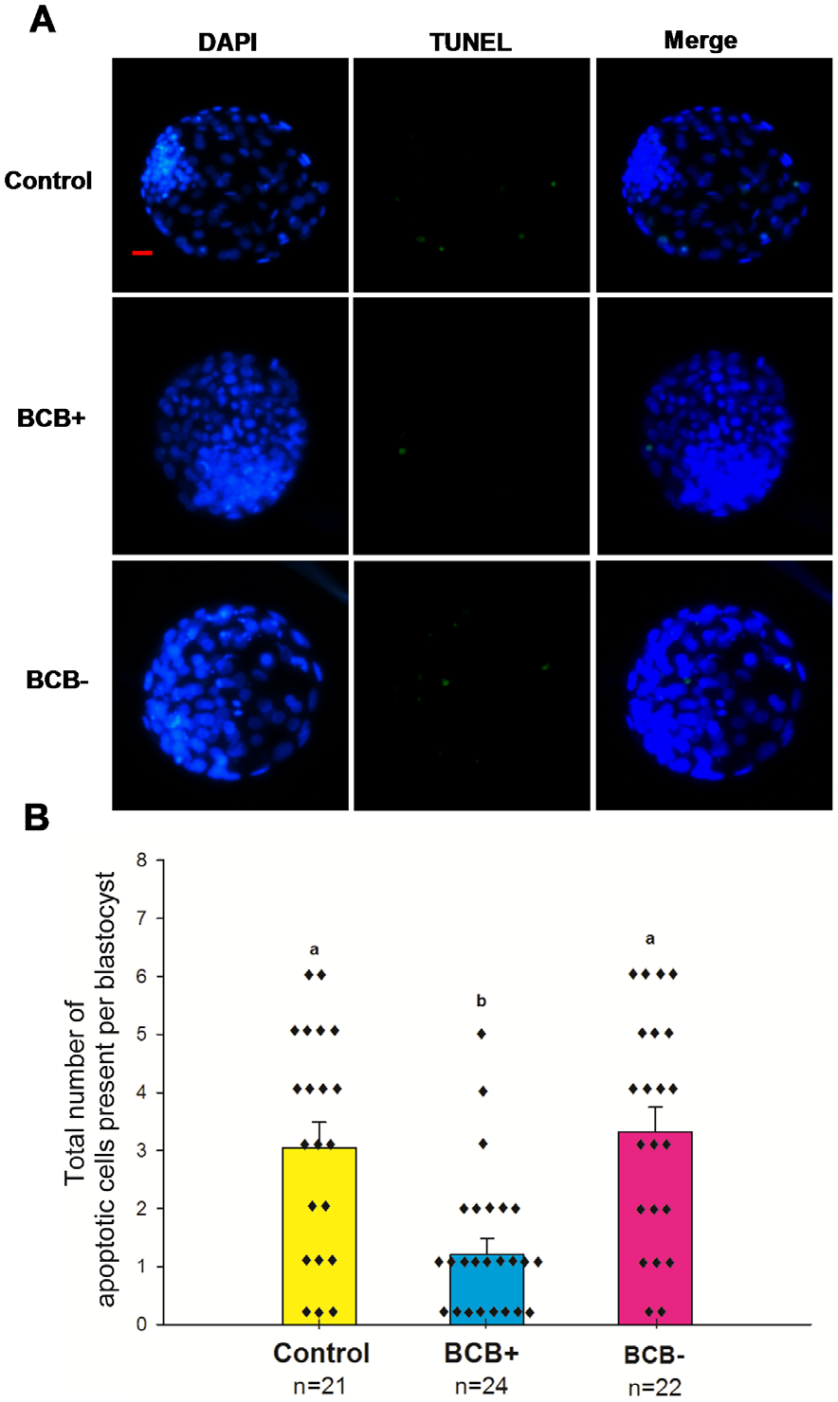
Incidence of apoptosis in blastocysts. (A) Representative photographs of TUNEL assay of blastocysts (green). Each sample was counterstained with DAPI to visualize DNA (blue). Original magnification was ×100. Bar = 20 µm. (B) Number of apoptotic cells in each blastocyst. Values with different superscripts differ significantly (P<0.05). n = 21, 24, and 22 in the control, BCB+, and BCB− groups, respectively.

Apoptosis is another criterion for evaluation of blastocyst quality. As shown in Figure 4, the apoptotic index was significantly lower in the BCB+ group than in the BCB− and control groups (P<0.05).

### Experiment 6. The mRNA and microRNA levels in SCNT blastocysts developed from differential oocytes

**Figure 5:**
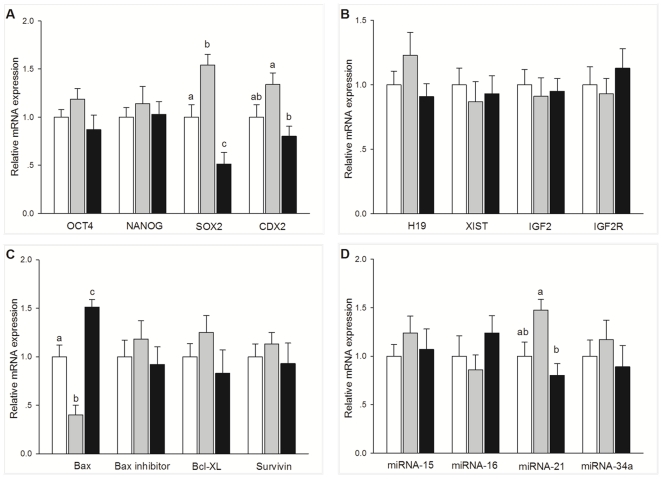
Relative abundance of apoptosis and development-related genes. Relative expression levels of development related genes (A), imprinted genes (B), apoptosis related genes (C), and microRNA (D) in single day 7 control (open bars), BCB+ (gray bars), and BCB− (black bars) blastocysts assayed by Quantitative real-time PCR and TaqMan RT-PCR. Values with different superscripts differ significantly (P<0.05); n = 5–8.

Relative expression levels of ICM- or TE-related genes, imprinted genes, and apoptotic-related genes were analyzed in blastocysts from control, BCB+, and BCB− groups using quantitative real-time PCR (Fig. 5). Expression of *SOX2* was significantly higher in BCB+ group compared with both BCB− and control group (P<0.05). The BCB− group was significantly lower in expression level of *SOX2* than the control group (P<0.05) (Fig. 5A). *CDX2* expression was lower in BCB− blastocysts than BCB+ blastocysts (P<0.05), but did not differ between the BCB− and control groups and between BCB+ and control groups (P>0.05) (Fig. 5A). The expression of *Bax* was significantly lower in BCB+ blastocysts than control and BCB− blastocysts (P<0.05) (Fig. 5C). *Bax* expression was significantly higher in BCB− blastocysts than control blastocysts (P<0.05) (Fig. 5C). There were no significant differences in the expression of *OCT4*, *NANOG*, *H19*, *XIST*, *IGF2*, *IGF2R*, *Bax* inhibitor, *Bcl-XL*, and *Survivin* among the three groups.

Relative expression levels of four microRNAs on blastocysts were analyzed by TapMan real-time RT-PCR (Fig. 5D). The expression level of microRNA-21 was significantly higher in BCB+ blastocysts than in BCB− blastocysts (P<0.05), but did not differ between the BCB− and control groups and between BCB+ and control groups (P>0.05).

## Discussion

Besides the donor cell and culture environment of embryos, the developmental competence (quality) of oocytes could be the most important limiting factor to somatic cloning efficiency. Many factors including age [11], [12], season [13], [14], nutrition [15], hormonal stimulation [16], storage temperature [17], [18], maturation environment [19], health of the follicle [20], follicle dominance [21], phase of follicular wave [22], and follicle size [23] have been found to affect oocyte quality and developmental potential. When COCs are aspirated with a syringe from ovaries, COCs may be recovered not only from antral follicles on the surface, but also from smaller antral follicles inside the ovary, which may be in the early stages of follicular development after antrum formation [8]. The follicular oocytes used in SCNT, which are usually recovered from ovaries of slaughtered animals, are commonly heterogeneous in quality and developmental competence [1]. Therefore, selection of good quality and developmentally competent oocytes is vital for the success of SCNT.

It has been proven that the use of BCB staining, based on the presence of active G6PDH in the growing immature oocyte, is efficient in selecting developmentally competent oocytes in various species, including cattle [1], [8], [9]. However, the effects of oocyte selection by BCB staining on the nuclear reprogramming capacity of oocytes, embryo quality, mRNA and microRNA expression, and even *in vivo* development of SCNT embryos have not yet been investigated. We explored the effects of oocyte selection by BCB staining on the *in vitro* and *in vivo* development of bovine SCNT embryos. In order to analyze the underlying mechanisms, we performed immunofluorescence staining on four epigenetic markers, differential staining of ICM and TE cells, apoptosis assays, qRT-PCR, and Taqman RT-PCR on SCNT embryos generated from BCB+, BCB− and control oocytes.

We found that the BCB+ oocytes had a significantly higher cloning efficiency than the BCB− and control oocytes. Bhojwani *et al.* [1] found that selection of developmentally competent oocytes through BCB staining enhanced blastocyst development rate after bovine nuclear transfer. However, the effects of oocyte selection by BCB staining on the *in vivo* development of SCNT embryos have not been investigated. Here, we found not only that BCB+ oocytes yielded increased blastocyst development rates, but also that the full-term development rate of bovine SCNT embryos was higher in the BCB+ group compared with the control and BCB− groups.

It is generally believed that the principal cause of low cloning efficiency is aberrant epigenetic nuclear reprogramming of the donor somatic cell by oocytes, which involves various epigenetic modifications. The acetylation and methylation of histone tails, the result of chromatin-modifying enzymes stored in oocytes, are major epigenetic modifications of the genome, which plays a significant role in the process of reprogramming, and ultimately affects the development of SCNT embryos [24]–[29]. Therefore, we analyzed the global acetylation levels of H3K9 and H3K18, and the global levels of histone H3 dimethylated at lysine 4 and 9 in bovine SCNT embryos and found that the acetylation levels of H3K9 and H3K18 were enhanced in BCB+ embryos at two-cell stage compared to BCB− ones. It is believed that increasing global acetylation of histones could alleviate transcriptional repression by facilitating chromatin remodeling and relieving methylated CpG sites [30], [31], and that hyperacetylation of histones could facilitate the access of various factors to nucleosomes [32]–[34]. Therefore, one of the ways in which oocyte selection by BCB staining improves the developmental potential of SCNT embryos *in vitro* and *in vivo* may be that the increased histone acetylation level facilitates chromatin remodeling and access of reprogramming-related factors to nucleosomes, alleviating transcriptional repression.

Strikingly, we also found BCB+ SCNT embryos at two-cell stage showed a higher level of methylation of histone H3 at K4 than BCB− ones. Research on histone methylation in embryos is very limited. It has been suggested that H3K4me2 might be closely related with zygotic gene activation in mice [35], and that SCNT embryos have lower levels of H3K4me2 compared with normally fertilized [36] or ICSI [37] control embryos at the two-cell stage. The methylation of histone H3 at K4 is correlated with activation of gene promoters. Therefore, the increased levels of H3K4me2 in BCB+ SCNT embryos perhaps facilitate zygotic gene activation in later stage embryos and that could contribute to genomic reprogramming of the somatic cell nuclei. Histone H3 dimethylated at lysine 9 is associated with euchromatin transcriptional repression and heterochromatin formation [38]. We found a similar pattern of H3K9me2 in control, BCB+, and BCB− SCNT embryos at the two-cell and blastocyst stage.

To further investigate the mechanisms behind the improved *in vivo* development of SCNT embryos after oocyte selection by BCB staining, we examined more closely the quality of bovine SCNT embryos developed from BCB+, BCB−, and control oocytes. We measured total, TE, and ICM cell numbers and calculated the ICM: TE ratio in blastocysts, which are commonly used criteria for assessment of blastocyst quality [39], [40]. We found that the number of total cells, TE cells, and ICM cells, and even the ratio of ICM: TE was significantly higher in BCB+ blastocysts compared with BCB− blastocysts. The total number of blastomeres and the ICM: TE ratio was also significantly higher in the BCB+ blastocysts than in control blastocysts. A previous study also demonstrated that the highest number of SCNT blastocyst nuclei was found in the BCB+ group with the lowest number in the BCB− group [1]. Recently, it was shown that BCB+ oocytes from prepubertal sheep produced blastocysts with a significantly higher number of total cells, ICM cells, and TE cells than BCB− oocytes [5]. To elucidate the mechanisms behind this, the relative expression levels of eight development-related genes *OCT4*, *NANOG*, *SOX2*, *CDX2*, *H19*, *XIST*, *IGF2*, and *IGF2R* were analyzed in SCNT blastocysts and up-regulated expression of *SOX2* and *CDX2* was found in the BCB+ group in comparison with the BCB− group. *SOX2* is a key regulator of pluripotency, and is important for maintaining ICM cell fate. Therefore, the up-regulated expression of *SOX2* in the BCB+ blastocysts may be associated with the higher number of ICM cells and increased ICM: TE ratio. The caudal-type homeodomain protein, CDX2, is the earliest known marker of the TE lineage. The up-regulated expression of *CDX2* in BCB+ blastocysts may correlate with the increase in TE cell number found in our study.

Besides the cell number of blastocysts, apoptosis is another criterion for evaluation of blastocyst quality. Here, the number of apoptotic cells was significantly lower in the BCB+ blastocysts than in the BCB− and control blastocysts. This suggests that BCB+ oocytes might generate high quality SCNT embryos by reducing cell death in the embryos. It was reported that the high rate of apoptosis in SCNT blastocysts was correlated with a decrease in the total cell number [40], which is consistent with our findings.

To suggest a cause of the reduced apoptosis rate, we further compared the relative expression levels of four apoptosis-related genes (*Bax*, *Bax* inhibitor, *Survivin*, *Bcl-XL*, and *Caspase-3*) and four microRNAs (miRNA-15, miRNA-16, miRNA-21, and miRNA-34a). We found *Bax* was down-regulated in the BCB+ blastocysts compared with BCB− and control blastocysts. The pro-apoptotic gene *Bax* is a positive regulator of apoptosis, so lower expression of *Bax* may contribute to the reduced apoptosis of cells in BCB+ blastocysts compared with those in BCB− and control blastocysts. Previous work found that developmentally important microRNAs, such as miRNA-127, are abnormally expressed in mouse embryos cloned by somatic cell nuclear transfer [41]. MicroRNA-21 has been classified as an oncogenic microRNA or biomarker that can indicate anti-apoptotic activity in various carcinomas [42]–[46]. The previous study found that the expression level of anti-apoptotic microRNA-21 was aberrantly lower in SCNT bovine embryos than in IVF embryos [47]. Here, we found microRNA-21 expression was up-regulated in the BCB+ blastocysts compared with BCB− blastocysts, which may contribute to the reduced apoptosis of cells in BCB+ blastocysts compared with those in BCB− blastocysts.

In conclusion, our study indicates that BCB+ oocytes yielded higher nuclear reprogramming capacity than BCB− and control oocytes, and benefited the *in vitro* and *in vivo* development of cloned bovine embryos. Therefore, BCB staining could be used as an effective method of selecting developmentally competent oocytes for nuclear transfer.

## Materials and Methods

### Ethics statement

The entire experimental procedure was approved and supervised by the Animal Care Commission of the College of Veterinary Medicine, Northwest A&F University. Bovine ovaries used in this study were obtained from Tumen abattoir and Zhongle abattoir, two local slaughterhouses located in Xi'An, China. A newborn female Holstein calf was obtained for nuclear donor cell cultures and Angus cows were used as recipients (Yangling Keyuan Cloning Co. Ltd).

### Chemicals

All chemicals and reagents were obtained from Sigma-Aldrich (St. Louis, USA) unless specifically stated otherwise. Disposable, sterile plasticware was obtained from Nunclon (Roskilde, Denmark).

### Oocyte collection and BCB staining

Oocyte collection was performed as described previously [48]. Briefly, bovine ovaries were kept in a thermos bottle with sterile saline at 20–25°C and were transported from the slaughterhouse to the laboratory within 4 h after the animal was killed. Cumulus–oocyte complexes (COCs) were aspirated from antral follicles (diameter, 2–8 mm) with a 12-gauge needle attached to a 10 ml syringe and were washed in PBS containing 5% (v/v) FBS. Only oocytes with a compact cumulus investment and with evenly granulated cytoplasm were selected for further culture and were designated as COCs.

**Figure 6:**
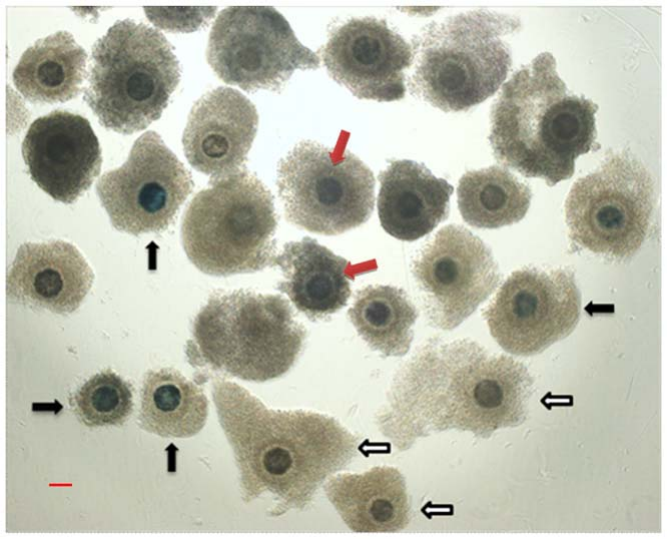
Representative photographs of differentially stained bovine COCs after exposure to BCB stain. BCB+ (blue cytoplasm), BCB− oocytes (colorless cytoplasm), and uncertain oocytes were directed by black, white, and red arrows, respectively. Bar = 100 µm.

After three washes in PBS containing 5% (v/v) FBS, COCs were exposed to 26 µM of BCB (B-5388, Sigma) for 90 min in 95% humidified air with 5% CO_2_ at 38.5°C. The concentration of 26 µM had earlier been found to be effective for cow, goat, pig, and mouse oocytes as it was supportive of a high rate of selected oocytes without apparent loss of viability. Oocytes of the control group were cultured immediately after selection without exposure to BCB. Following BCB staining, the COCs were washed twice in PBS. On the basis of their coloration (Fig. 6), oocytes were divided into BCB+ group (blue colored cytoplasm) and BCB− group (colorless cytoplasm). In order to achieve strict data, uncertain oocytes (uncertainty of staining status) were discarded in this study.

### *In vitro* maturation (IVM)

The COCs were washed twice in maturation medium (TCM-199, Gibco) containing 10% (v/v) FBS, 1 µg/ml 17β-estradiol, and 0.075 IU/ml Human Menopausal Gonadotropin and then were incubated for 22 h in the maturation medium in 95% humidified air with 5% CO_2_ at 38.5°C.

### SCNT, activation, and culture of SCNT embryos

Nuclear donor cell cultures were established from the ear skin of a newborn female Holstein calf as described previously [48]. Nuclear donor cells for SCNT were derived from passages 3 to 4 and cultured in serum-starved medium (0.5% FBS) for 2 days. SCNT, activation of reconstructed embryos, and culture of SCNT embryos were achieved as described previously [49]–[51]. Briefly, after IVM for 22 h, the cumulus cells of COCs were dispersed by vortexing in 1.5-ml centrifuge tubes. Only oocytes having an extruded first polar body and with uniform ooplasm were selected for enucleation. The first polar body and a small amount of surrounding cytoplasm were aspirated using a 20 µm inner diameter glass pipette in PBS microdrops containing 7.5 µg/ml cytochalasin B and 10% FBS. A single disaggregated donor cell was injected into the pre-vitelline space of the enucleated oocytes. The oocyte-cell fusion was performed using a pair of platinum electrodes connected to a micromanipulator in microdrops of Zimmermann's fusion medium, and a double electrical pulse of 35 V for 10 µs was used for fusion. Reconstructed SCNT embryos were kept in synthetic oviductal fluid (SOFaa) containing 5 µg/ml cytochalasin B for 2 h until activation. Activation of reconstructed embryos was performed in 5 µM ionomycin for 4 min followed by 4 h exposure to 1.9 mM dimethynopyridine (DMAP) in SOFaa. After activation, embryos were cultured in G1.5/G2.5 sequential media (Vitrolife AB, Gothenburg, Sweden). Droplets of 150 µl G1.5 were prepared in a 35-mm cell culture dish under mineral oil (20–30 embryos/microdrop). Embryos were transferred to G2.5 droplets on day 3 of culture (day 0 being the day of SCNT).

### Embryo transfer and pregnancy diagnosis

One quality day 7 SCNT blastocyst was transferred to the synchronized recipient uterine horn non-surgically ipsilateral to the corpus luteum 7 days after standing estrus. Pregnancy was detected by rectal palpation and ultrasonography at 40, 90 and 180 days of gestation (day 0 being the day embryos were transferred into recipients).

### Immunofluorescence staining of embryos

Immunofluorescence staining was carried out in accordance with the methods of our previous study [50]. Briefly, after embryos were fixed in Immunol Staining Fix Solution (Beyotime, Jiangsu, China) for 1 h, embryos were permeabilized with 0.2% Triton X-100 in PBS-PVA for 30 min. After three washes, they were blocked in the Immunol Staining Blocking Solution (Beyotime) for 12 h at 4°C and then incubated with the first antibodies for 12 h at 4°C. Anti-AcH3K9 (1∶500, Abcam, Cambridge, UK), anti-AcH3K18 (1∶500, Abcam), anti-H3K4me2 (1∶700, Beyotime), anti-H3K9me2 (1∶700, Beyotime), and anti-CDX2 (1∶200, BioGenex Inc., San Ramon, CA, USA) was diluted using Immunol Staining Primary Antibody Dilution Solution (Beyotime). Secondary antibodies were Alexa Fluor 488-labeled Goat Anti-Rabbit IgG (Beyotime) for AcH3K9, AcH3K18, and H3K4me2, H3K9me2 or Alexa Fluor 555-labeled Goat Anti-Mouse IgG (Beyotime) for CDX2. Finally, the DNA was stained with 4,6-diamidino-2-phenylindole (DAPI) (Beyotime) for 3 min, and samples were mounted on glass slides with a drop of Antifade Mounting Medium (Beyotime) and analyzed using a Nikon eclipse Ti-S microscope equipped with a 198 Nikon DS-Ri1 digital camera (Nikon, Tokyo, Japan). The intensity of AcH3K9, AcH3K18, H3K4me2, and H3K9me2 (green fluorescence) was analyzed using Image-Pro plus software (v6.0; Media Cybernetics, Silver Spring, MD, USA) and compared with that of DAPI signal (blue fluorescence). Using Image-Pro plus, images were converted to grayscale and inverted. After calibration of optical density (average cytoplasmic intensity were measured for normalization to background), all individual nuclei of embryos were outlined, and then integrated optical density (IOD) and area were measured. The average normalized fluorescence intensity for a single embryo was represented by ‘sum IOD/sum area’. Finally, AcH3K9, AcH3K18, H3K4me2, and H3K9me2 levels were divided by total DNA contents (DAPI total fluorescence intensities) to calculate normalized AcH3K9, AcH3K18, H3K4me2, and H3K9me2 quantities, respectively. To minimize the difference among embryos, all images were obtained with the same exposure times and adjustments of the microscope. During quantification of intensity of immunofluorescence using Image-Pro plus software, all parameters and adjustments were kept same. The experiments were replicated three times. In each replication, 10 to 15 embryos per group were processed. The level of histone acetylation or histone methylation of embryos was represented by mean value of embryos ± SEM. To quantify fluorescence intensity, the intensity levels of BCB+ and BCB− embryos were presented relative to the mean intensity level of control embryos.

### Apoptosis assays

Apoptosis assays were carried out using a DeadEnd Fluorometric TUNEL System (Promega, Madison, WI, USA) in accordance with the methods of our previous study [50]. Briefly, embryos were washed, fixed, and permeabilized as described for immunofluorescence staining. After equilibration in equilibration buffer, embryos were incubated with rTdT incubation buffer in the dark for 1 h at 37°C. The tailing reaction was terminated in 2× standard saline citrate for 15 min. Finally, the DNA was stained with DAPI (Beyotime). Samples were mounted on glass slides as described for immunofluorescence staining.

### Quantitative real-time PCR

The Cells-to-Signal™ Kit (Ambion, Austin, TX, USA) was used for embryos RNA isolation and RT reaction as described previously [50]. The RNA samples were treated with RNA-free Dnase I (Invitrogen) to digest residual genomic DNA. The mRNA levels were quantified using SYBR Premix ExTaq™ II (TaKaRa, Japan) on a CFX96 real-time PCR detection system (Bio-Rad, Richmond, CA) at the following thermal cycling conditions: 95°C for 1 min, followed by 40 PCR cycles of 95°C for 5 s, 55–60°C for 30 s, and 72°C for 30 s. The melting protocol was a step cycle starting at 65°C and increasing to 95°C with 0.5°C/5 s increments. The primer sequences are shown in Table S1 and were synthesized according to previous reports [52]–[55]. Housekeeping gene, *Histone 2a* (*H2A*) mRNA was employed as an internal standard for the analysis of relative transcript levels of each gene in various samples. Before the difference of genes expression were compared by quantitative PCR experiment, to evaluate the efficiency of amplification for internal control and target genes, templates were serial diluted and quantitative PCRs were processed to produce relative standard curve, the result showed that the efficiency of amplification for internal control were very close to target genes (96.6%–97%). Transcripts of target genes were quantified in three replicates and calculated relative to the transcription in every sample of *H2a*. The calibrator/control tissues for the real time PCR analysis are blastocysts from the control group. The specificity of the PCR reaction was confirmed by gel electrophoresis on a 2.5% agarose gel and by a single peak in the melt curve. The results of RT-PCR are presented as C_T_ value, where C_T_ was defined as the threshold cycle number of PCRs at which the amplified product was first detected. The 2^−ΔΔCT^ method [56] was used for relative quantification of target gene expression levels using the following formula:
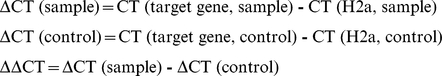



For ease of comparison, the average expression level of each gene from the control group was set as 1.

### TaqMan RT-PCR

All primers and the kit used in miRNA analysis was purchase from Applied Biosystems (Bedford, MA, USA). TaqMan RT-PCR used for relative quantification of miRNA-15, -16, -21, and -34 expressions was strictly conducted in accordance with the methods of a previous study [47]. H2A mRNA was employed as an internal reference.

### Experimental design and statistical analysis

#### Experiment 1

Collected oocytes were divided into BCB+ group, BCB− group, and control group. Oocyte maturation was indicated by first polar body emission. Oocytes maturation rates were evaluated after IVM for 22 h. SCNT was performed with oocytes from the three groups, *in vitro* development to two-cell and blastocyst stages was monitored at 48 and 168 h of culture, respectively (0 h being the time embryos were transferred to G1.5).

#### Experiment 2

Day 7 SCNT embryos developed from oocytes from the three groups were transferred to the synchronized recipient uterine horn non-surgically. Pregnancy rates and full-term development rate were determined to assess the *in vivo* development of SCNT embryos.

#### Experiment 3

Embryos of the three groups were collected at the two-cell and blastocyst stage for detecting the levels of AcH3K9, AcH3K18, H3K4me2, and H3K9me2.

#### Experiment 4

The total, TE, and ICM cell numbers in blastocysts of the three groups were estimated to assess the quality of embryos. The cell numbers in day 7 blastocysts were estimated by counting the total number of nuclei using DAPI. The number of trophectoderm (TE) nuclei was estimated using immunostaining for CDX2. The cell number of the ICM was assessed as the total number of nuclei minus the number of TE nuclei [50], [57].

#### Experiment 5

Apoptotic index in day 7 blastocysts was examined by TUNEL assay to assess the quality of embryos.

#### Experiment 6

The relative mRNA and microRNA levels in blastocysts were compared among the three groups.

### Statistical analysis

Outcomes were analyzed by one-way ANOVA and LSD tests using SPSS 18.0 software (SPSS Inc., Chicago, IL, USA). Differences were considered significant at P<0.05. Data were presented as mean ± SEM.

## Supporting Information

Table S1 **Primer sequences for real-time PCR.**
(DOC)
