# Supplementary material for: Oocytes Selected Using BCB Staining Enhance Nuclear Reprogramming and the In Vivo Development of SCNT Embryos in Cattle
Source: PLoS One. 2012 Apr 27;7(4):e36181. doi: 10.1371/journal.pone.0036181 (PMC3338625; doi:10.1371/journal.pone.0036181)
Supplement: Table S1 — Primer sequences for real-time PCR. (DOC) [file pone.0036181.s001.doc]

**Table S1. Primer sequences for real-time PCR**

| Genes | Primer sequences (5’-3’) | *T*_ann_^a^ (ºC) | Amplicon  size |
| --- | --- | --- | --- |
| OCT4 | F^b^: CCACCCTGCAGCAAATTAGC | 60 | 68 |
|  | R^c^: CCACACTCGGACCACGTCTT |  |  |
| NANOG | F: CGTGTCCTTGCAAACGTCAT | 60 | 66 |
|  | R: CTGTCTCTCCTCTTCCCTCCTC |  |  |
| SOX2 | F:GGTTGACATCGTTGGTAATTTATAATAGC | 60 | 88 |
|  | R: CACAGTAATTTCATGTTGGTTTTTCA |  |  |
| CDX2 | F: GCAAAGGAAAGGAAAATCAACAA | 60 | 120 |
|  | R: GGGCTCTGGGACGCTTCT |  |  |
| H19 | F: AGAGATGGTGCTACCCAGCTCA | 60 | 101 |
|  | R: TGTAGTGGTTCCAAAATGCAGC |  |  |
| XIST | F: AACCTCACGCCATTCCTCTG | 56 | 226 |
|  | R: GGGTAGGTGTTCCTCTTGAG |  |  |
| IGF2 | F: GCATCGTGGAAGAGTGTTGCTT | 60 | 102 |
|  | R: TCGTAGAGGCAGACACATCCCT |  |  |
| IGF2R | F: CTACGACCTGACCGAGTG | 60 | 95 |
|  | R: TGACAGCCTCCCAGTTG |  |  |
| Bax | F: TCTCCCCGAGAGGTCTTTTT | 57 | 151 |
|  | R:TGATGGTCCTGATCAACTCG |  |  |
| Bax inhibitor | F: CATGTGGTCACCCATTTCATTCA | 60 | 132 |
|  | R: AAATCCAGCCAGAAGTCCCAGTC |  |  |
| Bcl-XL | F: GGTATTGGTGAGTCGGATCG | 55 | 201 |
|  | R: CAAGACGACCCGAGTGAGAA |  |  |
| Survivin | F: CCTGGCAGCTCTACCTCAAG | 56 | 233 |
|  | R: TAAGTAGGCCAACACGAAAG |  |  |
| H2A | F: GTCTTGGAGTACCTGACCGC | 56 | 201 |
|  | R: AGTCTTCTTCGGGAGCAACA |  |  |

^a^ Annealing temperature. ^b^ Forward primer. ^c^ Reverse primer.
